# Walking Ability After Total Knee Arthroplasty: A Comprehensive Review

**DOI:** 10.7759/cureus.79577

**Published:** 2025-02-24

**Authors:** Nahum Rosenberg

**Affiliations:** 1 Specialists Center, National Insurance Institute - Israel, Haifa, ISR

**Keywords:** functional outcomes, orthopedic surgical techniques, postoperative rehabilitation, total knee arthroplasty, walking ability

## Abstract

Total knee arthroplasty (TKA) is a highly effective surgical intervention for alleviating pain and restoring function in patients with end-stage knee osteoarthritis. Walking ability, a critical measure of post-operative success, directly impacts patients' independence, mobility, and quality of life. This review comprehensively examines the factors influencing walking ability after TKA, encompassing pre-operative, surgical, and post-operative considerations. Pre-operative factors, such as patient demographics, functional status, psychological well-being, and comorbidities, significantly shape post-operative outcomes. Surgical techniques, including the choice of approach, implant design, alignment, and minimally invasive or robotic-assisted methods, also play a pivotal role in determining walking ability. Post-operative rehabilitation, particularly early mobilization, physical therapy, and exercise protocol adherence, is essential for optimizing recovery. Long-term outcomes reveal that, while most patients experience significant improvements in walking ability, some face persistent limitations due to age, comorbidities, or suboptimal surgical results. Emerging technologies, such as wearable devices, robotic-assisted surgery, and personalized rehabilitation programs, offer promising avenues for enhancing post-operative walking ability. Biological augmentation techniques, like platelet-rich plasma and stem cell therapy, are also being explored to improve tissue healing and functional recovery. This review underscores the importance of a multidisciplinary, patient-centered approach to maximize walking ability and overall satisfaction after TKA, synthesizing evidence from clinical studies, meta-analyses, and systematic reviews. Future research should focus on refining surgical techniques, advancing rehabilitation strategies, and integrating personalized medicine to improve outcomes.

## Introduction and background

Total knee arthroplasty (TKA) is one of the most successful orthopedic surgeries, with over 600,000 procedures performed annually in the United States alone [1]. The primary goals of TKA are to alleviate pain, improve joint function, and enhance the quality of life for patients with severe and symptomatic knee osteoarthritis. Among the various functional outcomes, walking ability is a key indicator of surgical success, as it directly impacts patients' independence, mobility, and overall well-being.

TKA is a widely used and highly successful surgical intervention for end-stage knee osteoarthritis, aiming to alleviate pain and restore function, ultimately improving the quality of life for affected individuals. The 10-year survivorship of the TKA prosthesis exceeds 90% [2], and it may even reach 99% [3]. Knee osteoarthritis, a degenerative joint disease, significantly impacts mobility and overall well-being, often leading to chronic pain, stiffness, and functional limitations. As the population ages and the prevalence of obesity rises, the demand for TKA grows, making it one of the most common elective orthopedic procedures performed worldwide. While TKA demonstrates high success rates in pain reduction and functional improvement, the degree to which patients regain their pre-operative walking ability remains an essential consideration [4].

Walking ability is critical to functional independence and overall quality of life. It influences an individual's ability to perform daily activities, participate in social engagements, and maintain employment. Restoring effective walking ability following TKA is, therefore, a primary objective of the procedure and subsequent rehabilitation. However, despite the overall success of TKA, some patients experience residual limitations in walking and other functional activities [5]. These limitations are related to a variety of factors, including patient-specific characteristics, surgical techniques, and the effectiveness of post-operative rehabilitation programs.

Walking ability after TKA is influenced by many factors, ranging from patient-specific characteristics to surgical and rehabilitation strategies. Despite the high success rate of TKA, some patients experience suboptimal walking ability postoperatively, leading to dissatisfaction and reduced quality of life [6].

This review provides a comprehensive overview of factors affecting walking ability after TKA, encompassing pre-operative assessment, surgical techniques, post-operative rehabilitation, and long-term outcomes. It examines the evidence on post-TKA walking recovery, exploring the multifaceted factors influencing it. The review discusses various assessment tools, from patient-reported outcomes to performance-based tests. It also addresses the impact of rehabilitation strategies, emphasizing comprehensive and individualized programs to improve walking outcomes. By understanding the factors contributing to optimal recovery, clinicians can tailor interventions to maximize patients' functional independence and quality of life after TKA.

## Review

Perioperative factors influencing walking ability

Accurately measuring and monitoring walking ability in patients following TKA is essential for guiding rehabilitation and evaluating the success of the surgical intervention. A variety of assessment tools, ranging from patient-reported outcome measures to performance-based tests, have been developed and validated for this purpose.

Patient-reported outcome measures, such as the Western Ontario and McMaster Universities Osteoarthritis Index, provide valuable insights into an individual's perceived functional status and satisfaction with the surgical outcome [7]. Performance-based tests, such as the six-minute walk test and stair climbing assessments, offer more objective measures of walking capacity and physical functioning [8].

Patient characteristics, such as age, BMI, and comorbidities, significantly influence post-TKA walking ability. Older patients and those with higher BMI often experience greater functional limitations and reduced walking capacity. Frailty and conditions like cardiovascular disease or diabetes can further impair recovery. Pre-operative functional status is crucial, as poorer baseline mobility often predicts slower and less complete rehabilitation progress. Psychological factors, including anxiety, depression, and fear of falling, can indirectly affect outcomes by impacting motivation and participation in rehabilitation. Furthermore, patient demographics, including age, gender, and BMI, are key determinants of walking ability after TKA. Older individuals and those with a higher BMI tend to experience slower recovery and poorer outcomes due to reduced muscle strength, joint stiffness, and the presence of comorbidities. Gender also plays a role, with women often reporting lower early functional scores and slower recovery compared to men. These variations underscore the importance of individualized rehabilitation programs tailored to each patient's specific needs and circumstances. Moreover, surgical techniques and the effectiveness of post-operative rehabilitation programs can significantly influence outcomes. Specifically, exercises, gait training, and targeted interventions to enhance lower limb strength, flexibility, and balance are particularly beneficial [9,10]. Emerging evidence suggests that perturbation training, involving controlled destabilizing forces, can further enhance balance and gait stability, contributing to improved walking ability after TKA. It's important to note that the application of perturbation training to TKA rehabilitation is still an emerging area. More research is needed to fully establish its effectiveness and determine the optimal protocols for this specific population [11].

The patient's pre-operative functional status strongly predicts post-operative walking ability. Patients with better pre-operative walking ability, muscle strength, and joint range of motion (ROM) are more likely to achieve better outcomes after TKA [8,12,13]. Conversely, patients with severe pre-operative disability, muscle atrophy, or limited ROM may experience slower recovery and reduced walking ability postoperatively [8,12,13].

Psychological factors, such as depression, anxiety, and low self-efficacy, have been shown to impact walking ability after TKA negatively. Patients with higher levels of pre-operative depression or anxiety are more likely to experience slower recovery, reduced adherence to rehabilitation, and poorer functional outcomes [14]. Therefore, pre-operative psychological assessment and intervention may improve post-operative walking ability and overall satisfaction.

The presence of comorbidities, such as cardiovascular disease, diabetes, and chronic obstructive pulmonary disease (COPD), can significantly affect walking ability after TKA. These conditions may limit patients' ability to participate in rehabilitation, reduce overall physical function, and increase the risk of post-operative complications. Pre-operative optimization of comorbidities is essential for improving post-operative walking ability [15]. While knee and functional scores improve significantly after TKA, the maximal walking ability (grade and distance) does not change considerably because there is a strong correlation between pre- and post-operative walking ability, suggesting that limitations in post-operative walking are primarily due to health comorbidities unrelated to the affected knee rather than the TKA itself [16].

Surgical factors, including the type of prosthesis, surgical approach, and intraoperative complications, also influence post-operative walking performance after TKA [17]. Different prosthetic designs can affect joint stability and kinematics, impacting gait patterns. The surgical approach can influence soft tissue damage and recovery time, indirectly affecting walking ability. Intraoperative complications, such as infection or nerve injury, can have significant negative consequences for post-operative recovery, including impaired walking ability.

The surgical approach used in TKA can influence post-operative walking ability. The most common methods include the medial parapatellar, subvastus, and midvastus approaches [18,19]. The medial parapatellar approach is widely used due to its excellent exposure of the knee joint. Potentially, it may result in more significant quadriceps muscle damage and slower recovery of walking ability [20]. The subvastus and midvastus approaches are associated with less quadriceps trauma and faster recovery of walking ability. Still, they may be technically more challenging and less suitable for obese patients or those with severe deformities [21].

Minimally invasive surgical (MIS) techniques have been developed to reduce soft tissue trauma, minimize post-operative pain, and accelerate walking ability recovery. MIS techniques involve smaller incisions and less disruption of the quadriceps muscle, which may lead to faster walking ability recovery and shorter hospital stays. However, the long-term benefits of MIS on walking ability and implant survival remain uncertain, and the technique requires a steep learning curve [22].

The design of the knee implant can also impact walking ability after TKA. Traditional fixed-bearing implants have been widely used and provide excellent long-term outcomes. However, mobile-bearing implants and high-flexion designs have been developed to improve ROM and walking ability. While some studies suggest that mobile-bearing implants may offer better functional outcomes [23], there is no reported conclusive evidence that supports this suggestion.

Proper alignment and balancing of the knee joint are critical for achieving optimal walking ability after TKA. Malalignment can lead to uneven wear, instability, and reduced walking ability. Computer-assisted surgery (CAS) and patient-specific instrumentation (PSI) have been introduced to improve the accuracy of alignment and balancing. While CAS and PSI have shown promise in reducing outliers in alignment, their impact on walking ability remains debated [24,25].

Role of rehabilitation in improving walking ability

Rehabilitation is critical in the recovery process following TKA, as it aims to restore mobility, strength, and overall functional independence. Comprehensive rehabilitation programs that incorporate physical therapy (PT), strengthening exercises, and gait training effectively improve walking ability after TKA [26,27].

Research has demonstrated that patients who engage in a structured rehabilitation regimen exhibit significant improvements in walking distance, stair climbing, and other functional measures compared to those who receive standard care or limited rehabilitation. By addressing factors such as muscle weakness, joint ROM, and balance impairments, rehabilitation interventions can help patients regain their walking capacity and achieve a higher level of independence in their daily activities [26].

Early mobilization is a cornerstone of post-operative rehabilitation after TKA. Early ambulation, typically within 24 hours of surgery, has been shown to reduce the risk of complications, such as deep vein thrombosis (DVT) and pulmonary embolism, and promote faster recovery of walking ability [28,29]. Early mobilization also helps prevent joint stiffness and muscle atrophy, which can negatively impact walking ability.

PT is essential for restoring walking ability after TKA. PT programs typically include exercises to improve strength, flexibility, balance, and gait. Strengthening exercises for the quadriceps, hamstrings, and hip abductors are particularly important for improving walking ability. Gait training, including assistive devices such as walkers or canes, is also a key component of PT and its primary effectiveness in the short-to-medium-term rehabilitation process, up to six months after surgery [30].

Continuous passive motion (CPM) devices are sometimes used postoperatively to improve ROM and reduce stiffness. While CPM may be beneficial in the early post-operative period, its impact on long-term walking ability is not clear. Combined post-operative treatment of CPM and PT may have a positive effect on the short-term rehabilitation process after surgery [31].

Adherence to post-operative rehabilitation is critical in determining walking ability after TKA. Non-adherence to PT exercises and recommendations can lead to suboptimal outcomes, including reduced walking ability and increased risk of complications. Strategies to improve adherence, such as patient education, goal setting, and regular follow-up, are essential for maximizing post-operative walking ability [32,33].

Long-term outcomes and walking ability

The timeline for functional recovery after TKA varies among patients, but most patients experience significant improvements in walking ability within the first six months postoperatively [34]. However, some patients may continue to improve for up to 12 months or longer [35]. Factors such as age, pre-operative functional status, and adherence to rehabilitation can influence the rate and extent of recovery [35].

Long-term walking ability after TKA is generally favorable, with most patients reporting significant improvements in pain, function, and quality of life. However, some patients may experience persistent limitations in walking ability, particularly those with comorbidities, poor pre-operative function, or suboptimal surgical outcomes. Long-term follow-up studies have shown that walking ability declines gradually over time, particularly in older patients and those with progressive joint degeneration [36], and also due to decreased quadriceps strength in the contralateral limb because of impaired gait patterns [37].

Patient satisfaction is closely linked to walking ability after TKA. Patients with good walking ability are likelier to report high surgery satisfaction. Conversely, patients with persistent limitations in walking ability may experience dissatisfaction and regret. Factors such as pain relief, functional improvement, and meeting pre-operative expectations are key determinants of patient satisfaction [38]. The published data reveal that about 20% of patients after TKA report unsatisfactory results from the surgery [39].

In some cases, patients may require revision surgery due to complications such as implant loosening, infection, or persistent pain [40]. Managing complications and timing revision surgery is critical for optimizing walking ability and overall outcomes.

Revision TKA is potentially associated with poorer outcomes than primary TKA, including reduced walking ability and lower patient satisfaction, but no substantial evidence supports this opinion [40].

Emerging technologies to improve walking ability

Wearable Technology

Wearable technology offers a promising avenue for enhancing walking ability after TKA. Devices like activity trackers and smart insoles provide real-time feedback on various aspects of gait, including step count, cadence, stride length, and even more nuanced biomechanical data [41]. This information empowers patients to actively participate in their recovery by monitoring their progress and identifying areas needing attention. For clinicians, these devices offer valuable objective data, complementing traditional assessment methods, and enabling more personalized and data-driven rehabilitation programs.

The real-time feedback provided by wearables can be particularly motivating, encouraging patients to adhere to their prescribed exercise regimens and maintain an active lifestyle. This feedback loop can lead to improved adherence to rehabilitation protocols, which is often a significant challenge in post-surgical recovery. Early studies suggest positive correlations between the use of wearable technology and improved walking ability, including increased walking distance and speed. However, more extensive research with larger sample sizes and more extended follow-up periods is needed to establish these technologies' long-term benefits and determine their optimal integration into rehabilitation programs [42].

Beyond simply tracking activity levels, some wearable sensors can detect deviations from normal gait patterns, alerting patients and clinicians to potential issues early on. This early detection can facilitate timely adjustments to rehabilitation strategies, preventing the development of compensatory movement patterns that could hinder long-term recovery. Furthermore, the data collected by wearables can be used to tailor rehabilitation programs to individual patient needs and track progress more objectively [43]. This personalized approach can optimize recovery and improve functional outcomes. While the initial results are promising, future research should focus on identifying the most effective types of wearable technology, optimal feedback modalities, and the best strategies for integrating these tools into comprehensive rehabilitation programs. This research will help unlock the full potential of wearable technology in maximizing walking ability and improving the quality of life for individuals after TKA.

Personalized rehabilitation

Personalized rehabilitation programs tailored to each patient's needs and goals can potentially optimize walking ability after TKA [44]. Using a digital health remote monitoring platform after TKA highlights the potential of technology to improve patient engagement and outcomes [44]. Individualized approaches, such as Goal Attainment Scaling, have shown promise in improving patient engagement and satisfaction with rehabilitation [44]. Furthermore, integrating technology, like digital health remote monitoring platforms, can enhance patient engagement and overall outcomes [45]. Advances in biomechanical assessment, imaging, and data analytics offer opportunities to create customized rehabilitation programs that address specific deficits and promote faster recovery [46]. For instance, wearable motion sensors can provide valuable data for tailoring rehabilitation exercises and monitoring progress. Personalized rehabilitation may also improve patient engagement and adherence, leading to better outcomes [35,46]. Studies exploring home-based rehabilitation with digital biofeedback systems suggest the potential of technology to enhance individualization and monitoring in rehabilitation programs. While further research is needed to establish personalized rehabilitation's efficacy in TKA fully, early findings suggest its potential to optimize walking ability and improve patient outcomes.

Future perspectives

The future of TKA holds significant promise for further enhancing walking ability and overall patient outcomes. Advances in surgical techniques, such as robotic-assisted surgery and PSI, are expected to improve precision in alignment and implant positioning, potentially leading to better functional recovery and long-term implant survival [24]. Emerging technologies, including wearable devices and artificial intelligence, offer opportunities for real-time monitoring of gait and adherence to rehabilitation, enabling personalized and adaptive post-operative care. Additionally, biological augmentation techniques, such as platelet-rich plasma (PRP) and stem cell therapy, are being explored to promote tissue healing, reduce inflammation, and improve muscle strength, which could further optimize walking ability [47]. Biological augmentation techniques are being studied as adjuncts to TKA to enhance tissue healing and functional recovery. While the evidence is still limited and further research is needed, these techniques promise to improve outcomes after TKA.

Integrating personalized medicine, tailored to individual patient characteristics and needs, will likely revolutionize rehabilitation protocols and surgical planning. Future research should focus on large-scale, long-term studies to validate these innovations and address disparities in access to advanced care. By incorporating these advancements, the field of TKA can continue to evolve, ultimately improving walking ability, patient satisfaction, and quality of life for individuals undergoing this transformative procedure.

Exoskeleton devices hold significant promise for improving walking ability after TKA. These devices can assist weakened muscles, improve joint stability, and ease ambulation, especially in the initial stage of post-operative rehabilitation [48,49]. Future research focuses on developing lighter, more adaptable exoskeletons that seamlessly integrate with the user's movements. Innovative exoskeletons incorporating sensors and artificial intelligence could personalize assistance levels based on real-time gait analysis, optimizing support and promoting faster recovery. Furthermore, advancements in battery technology and control systems are essential for enhancing the practicality and usability of these devices in everyday life. Integrating exoskeletons with telehealth platforms could enable remote monitoring and personalized rehabilitation programs, improving patient engagement and adherence [46]. While challenges remain in cost, accessibility, and user acceptance, ongoing research and development efforts suggest that exoskeletons could play an increasingly important role in restoring walking ability and improving quality of life after TKA.

## Conclusions

Walking ability is a crucial indicator of TKA success. Numerous factors affect post-TKA walking recovery, including patient demographics, pre-operative function, surgical techniques, and post-operative rehabilitation. While TKA implants have a medium to long-term survivorship rate of over 90%, patient satisfaction remains around 80%, primarily due to limitations in walking ability. Emerging technologies and personalized medicine offer promising avenues to bridge this gap by enhancing post-operative walking recovery and overall outcomes. A comprehensive understanding of these factors and a multidisciplinary approach to patient care are essential for optimizing ability and improving quality of life after TKA.
